# Evaluation of Rhubarb Supplementation in Stages 3 and 4 of Chronic Kidney Disease: A Randomized Clinical Trial

**DOI:** 10.1155/2014/789340

**Published:** 2014-09-11

**Authors:** Irfan A. Khan, Mohammad Nasiruddin, Shahzad F. Haque, Rahat A. Khan

**Affiliations:** ^1^Departments of Pharmacology, JNMCH, AMU, Aligarh 202002, India; ^2^Department of Medicine, JNMCH, AMU, Aligarh 202002, India

## Abstract

*Objective*. To evaluate the efficacy and safety of Rhubarb supplementation in patients of chronic kidney disease. *Material and Methods*. This study was a prospective comparative study conducted in patients of chronic kidney disease (stages 3 & 4) attending Renal Clinic of Department of Medicine, JN Medical College & Hospital, AMU, Aligarh. Patients were randomly divided into two interventional groups. Group I (Control) was given conservative management while Group II (Rhubarb) received conservative management along with Rhubarb capsule (350 mg, thrice daily) for 12 weeks. Haemogram and renal function tests were measured at 0, 4, 8, and 12 weeks of treatment. *Results*. There was progressive improvement in clinical features in both the groups after 12 weeks of treatment but Rhubarb group showed more marked improvement as compared to control group. Both groups showed gradual improvement in the biochemical parameters as compared to their pretreated values which was more marked in Rhubarb supplemented group. There was reduction in blood glucose, blood urea, serum creatinine, and 24 hour total urine protein (TUP). There was increase in haemoglobin, 24 hour total urine volume (TUV), and glomerular filtration rate (GFR). There was no statistical difference in two groups with respect to side effects (*P* > 0.05). *Conclusion*. Rhubarb supplementation improved the therapeutic effect of conservative management in stage 3 and stage 4 patients of chronic kidney disease.

## 1. Introduction

According to the National Kidney Foundation's Kidney Disease Outcomes Quality Initiative (K/DOQI) guidelines [1], chronic kidney disease is defined as kidney damage or glomerular filtration rate (GFR) <60 mL/min/1.73 m^2^ for 3 months or more, irrespective of cause. The prevalence of CKD in SEEK-India cohort was approximately 17.2% with ~6% having CKD stage 3 or worse [2]. Low protein diet-LPD (0.6 g/kg BW/day) as well as very low protein diet-VLPD (0.3 g/kg BW/day) decreases the accumulation of nitrogen waste products while maintaining an adequate nutritional status [3, 4]. The ideal treatment for CKD-ESRD (end stage renal disease) is renal replacement therapy (RRT) which includes renal transplantation and maintenance dialysis. Since these modalities are costly, required lifelong, not suitable for many patients, associated with many complications, and out of reach of 95–99% of patients, they are managed on conservative therapy [5].

Rhubarb belongs to genus* Rheum* in the family* Polygonaceae*. Important derivatives from Rhubarb are anthraquinones like rhein, emodin, and aloe emodin [6]. In CKD, these help in the elimination of nitrogenous products through the alimentary canal and regulation of water and electrolytes metabolism [7]. The abnormal expression of aquaporins (AQPs) could lead to less absorption of water in colon or more secretion of intestinal juice, suggesting that AQPs might be one kind of the effector molecules [8]. Chrysophanol and emodin inhibit the genetic transcription and translation of AQP2 gene. Rhubarb anthraquinones have the ability to downregulate AQP4 expression also [9]. In addition, effect of rhubarb was highly associated with the increasing serotonin levels and serotonin receptors in duodenum [10]. Rhein inhibits the transforming growth factor-beta 1 (TGF-*β*1) and fibronectin expression in renal tissue, thereby inhibiting extracellular matrix (ECM) deposition [11]. Emodin decreased the gluconeogenesis of renal tubular cells and diminished the ATP content of epithelial mitochondria. Both the Na^+^/K^+^-ATPase and Ca^2+^-ATPase activities of the epithelial cell were attenuated during the administration of emodin in an in vitro study [12]. In a prospective clinical trial conducted in 151 patients with chronic renal failure, the progression rate of renal failure was slowed in patients treated with rhubarb. There was increase in both the plasma albumin and transferrin level, pointing towards an improved nutritional status [7]. The aim of our study was to evaluate the efficacy and safety of rhubarb supplementation in patients of chronic kidney disease.

## 2. Material and Methods

### 2.1. Patients

The present study was conducted from June 2012 to September 2013 in patients of chronic kidney disease attending Renal Clinic of a tertiary care centre of north India. It was a randomized, prospective, double blinded, and parallel group study. The approval for the study was taken from Institutional Ethics Committee. The study is registered under Clinical Trial Registry of India with registration number CTRI/2012/09/002947 (registered on 03/09/2012). Written and informed consent was taken from all patients before enrolling in the study. The diagnosis of CKD was made on the basis of detailed clinical history, physical examination, and investigations.

### 2.2. Inclusion Criteria

Patients having CKD (stages 3-4), of age 20–60 years, and of either sex were included in the study.

### 2.3. Exclusion Criteria

Patients of end stage renal disease (ESRD), on dialysis, pregnant, terminally ill, immunocompromised, or of severe renal pathology such as malignancy were excluded from the study.

### 2.4. Sample Size (*n*)

*n* = (*z* ^2^/*e* ^2^)*pq*, where *z* = level of confidence interval at 95%, so *z* = 1.96; *e* = acceptable error; *p* = prevalence (prevalence assumed as 17.2% according to SEEK-India cohort study) [2]; and *q* = 1 − *p*. Hence, sample size (*n*) = [(1.96∗1.96)/(0.09∗0.09)]∗[0.172∗0.828] = 67.54. So, sample size of 68 is minimum required for each group. Taking into consideration a 15% dropout rate, 80 patients were recruited in each group. The power of the study using the study results (GFR) is 100%.

### 2.5. Study Design

**Figure 1:**
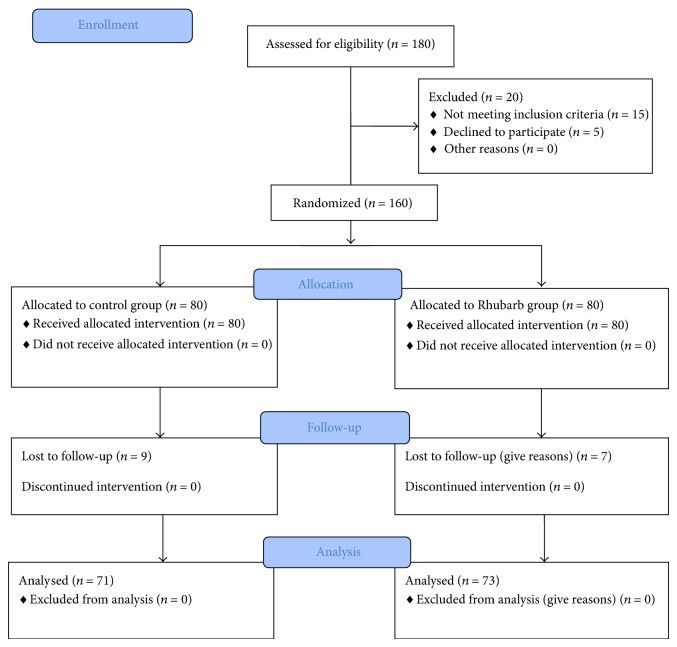
It shows recruitment, allocation, and follow-up of participants.

Out of 180 assessed patients, 160 patients were enrolled in the study. Fifteen patients (9 of Group I and 7 of Group II) failed to report on subsequent visits and were excluded from the study. Enrolled patients were randomized into two groups at a ratio of 1 : 1 using table generated by random allocation software. The randomization table had 20 subjects in each block to minimize the disparity between the two groups with respect to number of patients at any time of study. After final diagnosis, applying inclusion and exclusion criteria, patients were included in the study. Group I (Control) patients received conservative management of CKD along with placebo while Group II (Rhubarb) patients received conservative management of CKD along with Rhubarb capsule (350 mg) thrice daily (Figure 1). Both groups received treatment for 12 weeks. In conservative management treatment given was renal diet and telmisartan (40 mg OD). All the enrolled patients were regularly followed with haemogram and renal function tests at 0, 4, 8, and 12 weeks of treatment. The primary outcome in this study, that is, improvement in renal functions was assessed by blood urea, serum creatinine, 24 hour total urine protein (TUP), 24 hour total urine volume (TUV), and GFR while secondary outcomes were haemoglobin percent, fasting blood glucose, postprandial blood glucose, serum potassium, and serum calcium.

### 2.6. Safety Assessments

All adverse events experienced by a patient or observed by the investigator were recorded on standard ADR reporting forms of CDSCO at each visit. Rhubarb is reported to have laxative effect [7]. Adverse drug reaction's causality assessment was done using Naranjo's Scale [13] and severity assessment by Modified Hartwig & Siegel Scale [14]. A physical examination, including vital signs, was performed at the start of study and at each visit. Additional routine laboratory safety tests like liver function tests (LFT), ECG, and Chest X ray were performed wherever required.

### 2.7. Statistical Analysis

The values were expressed as mean ± SD. Statistical significance between pre- and posttreatment values in each group was calculated using Student's Paired* t*-test. Statistical significance between groups was calculated using unpaired* t*-test. *P* < 0.05 was considered significant. Statistical analysis was done using SPSS-20 software. The effect size calculated using improvement in serum creatinine was 0.2.

## 3. Result

71 (41 M, 30 F) patients mean aged 45 years (range 22–58 years) were of Group I and 73 (42 M, 31 F) patients mean aged 45 years (range 21–59 years) were of Group II. The distribution of patients was almost similar and no significant difference (*P* > 0.05) was seen between the groups. None of the patients in either group required dialysis and there was no mortality in either group. As per GFR (mL/min per 1.73 m^2^), patients belonged to stage 3 (19 and 20 in Group I and II resp.) and stage 4 (52 and 53 in Group I and II resp.) CKD in both the groups. The causes of CKD in groups I and II were diabetic nephropathy (45.07% and 43.83%), hypertensive nephropathy (18.30% and 19.17%), chronic glomerulonephritis (11.26% and 9.58%), tubulointerstitial nephritis (8.45% and 6.84%), autosomal dominant polycystic kidney disease (4.22% and 5.47%), and unknown cause (12.67% and 15.06%).

In the present study, the clinical features found in the patients at admission were anorexia, nausea, vomiting, weakness, weight loss, headache, pruritus, swelling over body, oliguria, anaemia, hypertension, and dyspnoea. The clinical features were almost similar at 0 week in both the groups. There was gradual improvement in clinical features in both the groups after 12 weeks of treatment but it was more marked in Rhubarb group.

There was progressive decrease in both systolic and diastolic blood pressure towards normal in both the groups. As compared to control group, Rhubarb group showed significant (*P* < 0.05) reduction in both systolic and diastolic blood pressure after 12 weeks of treatment (Table 1).

The total leucocyte count (TLC), differential leucocyte count (DLC), and platelet count remained within normal limits at the end of 12 weeks of treatment in both the groups.

There was progressive improvement in various biochemical parameters in both the groups; Rhubarb group showed maximum improvement. As compared to control group, Rhubarb group showed significant increase in haemoglobin percent (*P* < 0.05), decrease in fasting and postprandial blood glucose (*P* < 0.01), decrease in blood urea (*P* < 0.05), and decrease in serum creatinine (*P* < 0.05) at 12 weeks. There was decrease in serum potassium in both the groups which was significant (*P* < 0.05) in Rhubarb group as compared to control. There was significant increase in serum calcium (*P* < 0.01), decrease in TUP (*P* < 0.05), increase in TUV (*P* < 0.001), and increase in GFR (*P* < 0.001) after 12 weeks of treatment in Rhubarb group as compared to control group (Table 2).

The adverse drug reactions occurrence was not significantly different between control and Rhubarb groups. According to Modified Hartwig and Siegel Scale, the adverse drug reactions were mild (no hospitalization, no change of therapy, and no additional treatment) in severity in both the groups. No adverse event was of acute onset (within 60 minutes). On Naranjo's Scale, the ADRs were possible (scores = 1–4) in 12 cases and probable (scores = 5–8) in 11 cases with control group while possible (scores = 1–4) in 14 cases and probable (score = 5–8) in 8 cases with Rhubarb group (Table 3).

## 4. Discussion

Chronic kidney disease (CKD) is an emerging chronic disease globally due to rapidly increasing incidence of diabetes and hypertension worldwide [15, 16]. CKD leads to premature morbidity and mortality and hampers quality of life. In India, CKD is a major problem for both health sector and economy. More than 100,000 new patients enter RRT annually in India [17]. Because of meagre resources, only 10% of Indian ESRD patients receive any RRT. The monthly cost of hemodialysis is $300, whereas CAPD costs $600. The cost of transplant is $8900 in the first year, which declines later to $3000 annually. Among the RRT options, renal transplant is the preferred choice as it is cost effective and offers better quality of life but still only a fraction of Indians can afford it [17].

Conservative management is very important to prevent CKD and to prevent progression of CKD to ESRD. It delays the progressive deterioration of renal function. It provides only symptomatic relief. So, newer treatment modalities are being searched which can halt nephron damage, delay the development of ESRD and are cost effective.

Previous studies have reported beneficial effect of rhubarb in CKD patients [18, 19]. Rhubarb contains various phytoconstituents among which rhein and emodin are important because of their beneficial effect in CKD. Rhein inhibit cell hypertrophy and extracellular matrix (ECM) accumulation by decreasing the transforming growth factor-beta 1 (TGF-*β*1) and fibronectin expression in renal tissue [20]. TGF-*β*1 stimulates the glucose uptake in mesangial cells through upregulation of GLUT 1 expression. Emodin has inhibitory effect on the expression of c-myc mRNA and hence on cell cycle downregulation in cultured rat mesangial cells, which might be the reason why emodin inhibits mesangial cell proliferation [21]. Rhubarb suppresses the production of various cytokines from macrophages and human mesangial cells [22, 23]. Rhubarb also has laxative effect which increases excretion of nitrogenous wastes from the body [7, 8]. These might be the probable mechanisms for beneficial effects of rhubarb in our study.

Rhubarb showed beneficial effects in CKD patients at a dose of 1000 mg/day [19]. So, Rhubarb dose used in our study was 350 mg TDS daily.

According to Ye et al., there was no side effect of rhubarb administration at a dose of 8–12 g/day for 3 weeks in 30 patients of CKD [18]. So, the ADRs might be the manifestations of underlying renal pathology or due to other coadministered drugs.

The findings in our study are in accordance with those reported in previous studies. The drawback of this study is its limited duration of study. Longer duration of follow-up is needed in further studies to see the long term effect of rhubarb in CKD patients.

So, supplementation of Rhubarb along with conservative management produces improvement in clinical features and in biochemical parameters and is safe in patients of chronic kidney disease.

## 5. Conclusion

Rhubarb supplementation improved the therapeutic effect of conservative management in stage 3 and stage 4 patients of chronic kidney disease.

## Figures and Tables

**Table 1:** Blood pressure in control and Rhubarb groups before and after 12 weeks of treatment.

Serial number	Parameter	Group	0 week mean ± SD	12 weeks mean ± SD	% change after 12 weeks	95% confidence interval
1	SBP (mm Hg)	I	150.40 ± 17.62	136.62 ± 16.45^b^	(−) 9.16%	22.81 to 32.90
		II	152.97 ± 20.60	132.60 ± 8.79^b1^	(−) 13.31%	31.93 to 44.30

2	DBP (mm Hg)	I	87.32 ± 10.43	85.98 ± 9.65	(−) 1.53%	12.43 to 22.92
		II	88.02 ± 12.40	84.73 ± 9.31^b1^	(−) 4.40%	12.97 to 23.30

Values are mean ± SD; *P* < 0.05 was considered significant; ^b^ *P* < 0.01 compared to 0 week value of respective group; ^1^ *P* < 0.05 compared to control group. I: Control; II: Rhubarb; SBP: Systolic blood pressure; DBP: Diastolic blood pressure; (−) decrease.

**Table 2:** Haemogram and renal function tests in control and Rhubarb groups before and after 12 weeks of treatment.

Serial number	Parameter	Group	0 week mean ± SD	12 weeks mean ± SD	% change after 12 weeks	95% confidence interval
1	Hb% (g/dL)	I	7.91 ± 1.93	8.91 ± 1.48^c^	(+) 12.64%	(−) 1.24 to (−) 0.75
		II	7.87 ± 2.06	9.05 ± 1.58^c1^	(+) 14.99%	(−) 1.51 to (−) 0.85

2	FBG (mg/dL)	I	130.05 ± 42.90	113.78 ± 14.31^c^	(−) 12.51%	8.88 to 23.64
		II	132.60 ± 45.55	107.20 ± 18.03^c2^	(−) 19.15%	14.97 to 35.82

3	PPBG (mg/dL)	I	184.95 ± 61.17	157.56 ± 23.20^c^	(−) 14.80%	17.38 to 37.40
		II	182.30 ± 62.05	147.65 ± 15.46^c2^	(−) 19.00%	23.17 to 46.11

4	B. Urea (mg/dL)	I	107.16 ± 35.85	79.78 ± 24.79^b^	(−) 25.55%	21.83 to 32.92
		II	108.89 ± 42.65	72.25 ± 20.89^c1^	(−) 33.64%	30.97 to 42.30

5	S.Cr. (mg/dL)	I	4.44 ± 1.64	3.33 ± 1.37^c^	(−) 25.00%	0.86 to 1.37
		II	4.06 ± 2.08	2.82 ± 1.11^ c1^	(−) 30.54%	0.91 to 1.55

6	K^+^ (mEq/L)	I	4.87 ± 0.49	4.63 ± 0.41^a^	(−) 4.92%	0.27 to 0.41
		II	4.84 ± 0.44	4.42 ± 0.48^b2^	(−) 8.67%	0.26 to 0.57

7	Ca^2+^ (mg/dL)	I	8.65 ± 1.05	8.89 ± 1.00^a^	(+) 2.77%	(−) 0.53 to 0.07
		II	8.72 ± 1.01	9.38 ± 0.90^b2^	(+) 7.56%	(−) 0.93 to (−) 0.38

8	TUP (g/day)	I	3.03 ± 1.29	2.43 ± 0.97^b^	(−) 19.80%	0.35 to 0.85
		II	3.18 ± 1.57	2.12 ± 0.65^c1^	(−) 30.18%	0.83 to 1.09

9	TUV (mL/day)	I	1454.36 ± 221.53	1736.76 ± 176.04^c^	(+) 19.41%	(−) 333.40 to (−) 230.38
		II	1451.69 ± 303.74	1870.14 ± 258.78^c3^	(+) 28.82%	(−) 467.28 to (−) 369.61

10	GFR (mL/min)	I	19.0 ± 1.17	23.3 ± 1.63^b^	(+) 22.6%	5.28 to 9.73
		II	19.1 ± 2.37	28.0 ± 3.51^c3^	(+) 46.5%	5.99 to 15.31

Values are mean ± SD; *P* < 0.05 was considered significant; ^a^ *P* < 0.05, ^b^ *P* < 0.01, ^c^ *P* < 0.001 compared to 0 week value of respective group; ^1^ *P* < 0.05, ^2^ *P* < 0.01, ^3^ *P* < 0.001 compared to control group. I: control; II: Rhubarb; Hb%: haemoglobin percent; FBG: fasting blood glucose; PPBG: postprandial blood glucose; B. urea: blood urea; S.Cr.: serum creatinine; K^+^: serum potassium; Ca^2+^: serum calcium; TUP: 24 hour total urine protein; TUV: 24 hour total urine volume; GFR: glomerular filtration rate; (−) decrease; and (+) increase.

**Table 3:** Comparison of adverse drug reactions (ADRs) between control and Rhubarb group.

Serial number	ADR recorded	Control (*n* = 71)	Rhubarb (*n* = 74)	Significance (2-tailed)
1	Nausea	5	4	0.494
2	Vomiting	4	3	0.442
3	Diarrhea	5	3	0.275
4	Constipation	0	1	0.497
5	Anorexia	4	2	0.719
6	Excessive thirst	0	1	0.245
7	Abdominal pain	1	1	1.000
8	Muscle and joint pain	0	1	1.000
9	Headache	3	2	0.366
10	Rashes	0	2	1.000
11	Altered taste	0	1	1.000
12	Weakness	1	0	0.497
13	Frequent urination	0	2	1.000

*P* < 0.05 was considered significant. Fisher's exact test was applied.
